# Méningite après rachianesthésie

**DOI:** 10.11604/pamj.2016.24.139.8390

**Published:** 2016-06-10

**Authors:** Issam Mouchrif, Adnane Berdaii, Ismail Labib, Moustapha Harrandou

**Affiliations:** 1 Service de Réanimation Mère et Enfant, CHU Hassan II, Fès, Maroc

**Keywords:** Asepsie, méningite, rachianesthésie, Asepsis, meningitis, spinal anesthesia

## Abstract

Les méningites sont des complications rares mais graves des anesthésies péridurales et des rachianesthésies. Les méningites bactériennes sont essentiellement dues à des cocci à Gram positif, ce qui sous-entend une contamination exogène pouvant être le témoin d'une faute d'asepsie. L’évolution est le plus souvent favorable sous traitement, mais au prix d'un accroissement des dépenses de santé et parfois d'importantes séquelles neurologiques. Nous présentons un cas de méningite bactérienne au décours d'une rachianesthésie effectué pour césarienne.

## Abstract (en)

Meningitis is a rare but serious complication of epidural and spinal anesthesia. Bacterial meningitis is mainly caused by Gram-positive cocci, implying an exogenous contamination which suggests a lack of asepsis. The evolution is usually favorable after treatment, but at the expense of increased health care costs and, sometimes, of significant neurological sequelae. We report a case of bacterial meningitis after spinal anesthesia for caesarean section.

## Introduction

Plusieurs complications peuvent survenir après une anesthésie locorégionale. Les méningites sont des complications rares des rachianesthésies et des anesthésies péridurales [1], dont le pronostic est étroitement lié à la précocité du diagnostic, à l'identification du germe, et au traitement. Nous rapportons un cas de méningite bactérienne après une rachianesthésie.

## Patient et observation

Une parturiente âgée de 21 ans, est proposée pour une césarienne urgente indiquée pour une disproportion foeto-pelvienne. En préopératoire, la patiente était apyrétique à 37,3^°^C. Le reste de l'examen était sans particularités. La leucocytose était à 9200 / mm^3^. L'intervention s'est déroulée sous rachianesthésie réalisé avec une aiguille de 25 G et de la bupivacaine isobare à 0,5%, les règles d'asepsie ont été respectées. La parturiente avait reçu une antibioprophylaxie à base d'amoxicilline protégée. L’évolution était marquée, vingt-six heures après, par l'apparition des céphalées et de vomissements. L'examen clinique trouvait une patiente obnubilée avec un GCS à 13, stable sur le plan hémodynamique (une pression artérielle à 130/80 mm hg, une fréquence cardiaque à 100 battements/minute), eupnéique avec un syndrome méningé franc et une température à 39^°^C. La numération formule sanguine montrait des leucocytes à 21 460 mm^3^ (89 P.100 de neutrophiles), des plaquettes à 663 000 mm^3^, une hémoglobine à 9,7 g/dl. L'ionogramme sanguin était dans les limites de la normale. La fonction rénale, le bilan hépatique, la lacticodéshydrogénase et le bilan de crase étaient normaux. Une TDM cérébrale était réalisée, revenant sans anomalies. La ponction lombaire trouvait un LCR trouble avec 3200 EB / mm^3^ à prédominance PNN altérés, glucorachie à 0,6 mmol/l (glycémie concomitante à 3,7 mmol/l), albuminorrachie à 2,03g/L, la recherche des antigènes solubles et la culture étaient revenus négatifs. La patiente a été mise sous vancomycine et ceftriaxone à dose méningée pendant cinq jours puis sous amoxicilline avec une durée totale de dix jours. L’évolution était favorable avec une apyrexie obtenue au bout de 24 heures et la ponction lombaire réalisée après 48 heures était normale.

## Discussion

La survenue d'une méningite après une rachianesthésie est un événement rare dont l'incidence est estimée à moins de 4,5 cas pour 100 000 actes [2, 3]. La symptomatologie clinique des méningites après anesthésie locorégionale rachidienne est assez univoque. L'association de céphalées, d'hyperthermie, de signes méningés francs et éventuellement de troubles de la conscience au décours d'une rachianesthésie doit faire immédiatement suspecter le diagnostic de méningite et conduire à la réalisation d'une ponction lombaire exploratrice [1, 4]. Les germes les plus fréquemment rencontrés dans ce cas sont des cocci à Gram positif, comme le Streptococcus Sanguis, et plus rarement des bacilles à Gram négatif. Les méningites peuvent être d'origine infectieuse; le plus souvent bactérienne, ou dues à une irritation locale: méningite aseptique. Il est impossible de trancher entre une méningite aseptique et méningite décapitée [5, 6]. Les méningites aseptiques sont dues à une réaction méningée lors de l'introduction des substances ou de corps étranger irritants dans l'espace sous arachnoïdien [7]. Le diagnostic formel de méningite bactérienne est posé par la mise en évidence de germes, soit à l'examen direct, soit après culture.

Plusieurs modes de contamination ont été décrits. La contamination exogène est suspectée lorsque l'examen du LCR met en évidence des saprophytes cutanés, ce qui fait suspecter une mauvaise antisepsie de la peau, ou des saprophytes de l'oropharynx. Plusieurs cas de contamination du LCR par des germes provenant de l'oropharynx de l'anesthésiste ont été décrits avec, pour certains, la preuve de la contamination par identification génétique du germe pathogène et des souches présentes dans l'oropharynx de l'anesthésiste [8, 9]. L'antisepsie cutanée doit être effectuée en quatre temps (lavage, rinçage, séchage puis désinfection) à fin de réduire les risques de contamination d'origine cutanée. Par ailleurs, le port de gants stériles, de masque et de calot a fait la preuve de son efficacité et est préconisé par la Société française d'anesthésie et de réanimation et par l'American Society of Anesthesiologists [10]. L'autre mode de contamination est la voie endogène, par greffe septique, La contamination du LCR serait favorisée par des effractions vasculaires. L’évolution de l'infection est habituellement favorable après traitement antibiotique adapté au germe en cause. Cependant, l'existence de séquelles neurologiques permanentes a été décrite [11].

## Conclusion

La survenue d'une méningite au décours d'une rachianesthésie est exceptionnelle. Cependant, l’éventualité de décès ou de séquelles graves ne doit pas faire négliger ce risque. Le diagnostic, lorsqu'il est évoqué devant un syndrome méningé fébrile, doit conduire à la réalisation d'une ponction lombaire exploratrice. Les sources de contamination sont très fréquemment exogènes et les germes proviennent le plus souvent de la flore cutanée du patient ou de la flore ORL du médecin anesthésiste. Le diagnostic et le traitement doivent être précoces. La découverte, lors de la ponction lombaire, de cocci à Gram positif est en faveur d'une contamination exogène, qui peut mettre en cause la responsabilité directe de l'anesthésiste-réanimateur. Conformément aux bonnes pratiques cliniques, les mesures d'hygiène élémentaires doivent être scrupuleusement appliquées.
